# A 7-day high-PUFA diet reduces angiopoietin-like protein 3 and 8 responses and postprandial triglyceride levels in healthy females but not males: a randomized control trial

**DOI:** 10.1186/s40795-018-0262-7

**Published:** 2019-01-06

**Authors:** Sepideh Kaviani, Caroline M. Taylor, Jada L. Stevenson, Jamie A. Cooper, Chad M. Paton

**Affiliations:** 1 Department of Foods and Nutrition, University of Georgia, Athens, GA USA; 2 Department of Food Science and Technology, Department of Foods and Nutrition, University of Georgia, 100 Cedar St., Athens, GA 30602 USA; 3 Texas Christian University, Fort Worth, TX USA

**Keywords:** Polyunsaturated fatty acid, Saturated fatty acid, High fat, Triglyceride, Angiopoietin-like proteins, ANGPTL3, ANGPTL8, ANGPTL4

## Abstract

**Background:**

Polyunsaturated fatty acids (PUFAs) have beneficial effects on hypertriglyceridemia although their effect on angiopoietin-like proteins (ANGPTLs), specifically ANGPTL3, ANGPTL4 and ANGPTL8 is unknown.

**Objective:**

To determine whether a high-PUFA diet improves postprandial triglyceride (TG) levels through reducing ANGPTL responses following high saturated fat (SFA) meals.

**Methods:**

Twenty-six adults were randomized into a PUFA diet (*n* = 16) or a control diet group (*n* = 10). Participants completed a pre-diet visit (v1) where they were given two SFA-rich, high-fat meals. Blood draws were taken at fasting and every 2 h postprandially for a total of 8 h. After v1, participants completed a 7d diet of the same macronutrient proportions (50% carbohydrate, 35% fat, 15% protein) but with different fatty acid (FA) compositions (PUFA = 21% of total energy from PUFAs vs. Control = 7% of total energy from PUFA). All participants then completed the post-diet visit (v2) identical to v1.

**Results:**

In the PUFA group, females, but not males, reduced TG concentrations (Area under the curve (AUC): 141.2 ± 18.7 vs. 80.7 ± 6.5 mg/dL/h, *p* = 0.01, for v1 vs. v2, respectively). Fasting and postprandial AUC levels of ANGPTL3 and 8, but not ANGPTL4, also decreased from v1 to v2 in PUFA females, but not males. No changes from v1 to v2 were seen in either sex in the control group.

**Conclusions:**

A PUFA-rich diet improves TG levels in response to high-SFA meals with reductions in ANGPTL3 and ANGPTL8. PUFAs may be more protective against hypertriglyceridemia in females, compared to males since no diet effect was observed in males.

**Trial registration:**

NCT02246933.

**Electronic supplementary material:**

The online version of this article (10.1186/s40795-018-0262-7) contains supplementary material, which is available to authorized users.

## Background

Hypertriglyceridemia is a form of dyslipidemia that is categorized by elevated blood triglyceride (TG) concentrations and affects nearly 30% of the US population [1–3]. High plasma TG levels are associated with type 2 diabetes mellitus and obesity, possibly due to lipid-induced insulin resistance, glucose intolerance, and increased very low-density lipoprotein (VLDL) production [1, 2, 4, 5]. Elevated postprandial blood TGs are a public health concern since they have shown to be an independent risk factor for cardiovascular disease and atherosclerosis [4, 5].

In addition to chronic high fat (HF) *diets*, previous studies have shown the impact of individual HF *meals* on cardiovascular disease (CVD) risk by measuring markers such as inflammatory factors, thrombosis, and blood pressure responses [6–8]. The majority of the detrimental effects of dietary fat consumption appears to be from saturated fat with mono- and polyunsaturated fat appearing to be protective, or at least not harmful. A diet high in long chain omega-3 polyunsaturated fatty acids (PUFA), specifically α-linolenic acid (18:3n3), eicosapentaenoic acid (EPA) (20:5n3) and docosahexaenoic acid (DHA) (22:6n3), is linked with reduced fasting blood TG levels, increased high density lipoprotein (HDL) cholesterol, and decreased low density lipoprotein (LDL) cholesterol [9–12]. Contrary to the beneficial role of PUFAs on plasma TG, consuming saturated fatty acids (SFAs) has been shown to increase LDL cholesterol, decrease insulin sensitivity and may promote inflammation if consumed as part of a hypercaloric diet, therefore increasing the risk of CVD [13–15].

Some of the effects of dietary PUFAs may be from increased oxidation compared to SFAs, however the mechanism by which this occurs is not known [16, 17]. A newly identified model for the systemic regulation of lipid metabolism is through three members of the eight-member family of angiopoietin-like proteins (ANGPTLs) including ANGPTL3, − 4, and − 8 and their tissue specific regulation of lipoprotein lipase (LPL) activity [18]. ANGPTL3, for instance, decreases the clearance of VLDL triglyceride through constraining LPL activity [19], directly affecting adipocytes to activate lipolysis [20]. A consequent increase in the release of free fatty acid (FFA) and glycerol from adipocytes as a result of ANGPTL3 functioning indicates that it might be an important regulatory agent in lipid metabolism [21].

Several genetic studies have shown distinct effects of common variants in the ANGPTL3, ANGPTL4 and ANGPTL8 genes on plasma lipid levels [22, 23]. Functional studies have revealed that these three ANGPTLs influence plasma lipid levels by inhibiting the activity of extracellular lipases, including the structurally related LPL, as well as hepatic, endothelial and pancreatic lipases during fasting and fed states [18, 19, 22–28]. Hence, these three ANGPTLs are postulated to generate a framework for how TG trafficking is regulated given their LPL-inhibitory roles [25]. Consequently, it is reasonable to conclude that deficiency of any of these ANGPTLs could result in hypotriglyceridemia [25]. Altogether, it would be sensible to hypothesize that replacing dietary SFAs with PUFAs may aid in reducing plasma TG levels following a possible diminished ANGPTL3, 4, and 8 response, especially after acute consumption of SFA-rich meals. However, whether these ANGPTLs are affected differently by various dietary fatty acids in humans remains unknown.

The purpose of this study was to 1) determine whether a high PUFA diet could mitigate the effects of a high SFA meal through reduced postprandial TG levels, and 2) to explore the mechanisms behind any plasma changes in plasma TG levels by examining the changes in ANGPTL3, − 4, and − 8. Based on previous work on the impact of dietary FA composition on plasma TG levels, we hypothesized that consuming a high PUFA diet may aid in reducing plasma TG levels. We found that postprandial TG levels were decreased following a PUFA-rich diet. In order to examine the possible mechanisms for this reduction, we measured changes in plasma ANGPTL3, − 4, and − 8 levels. Despite the fact that there are limited data on the mechanisms of how FA composition changes in the diet can lead to alterations in plasma TG levels, we were able to provide clear evidence that a PUFA-rich diet will decrease postprandial TG levels in women.

## Methods

### Study design

As previously reported [29], a single-blinded randomized control feeding trial with a parallel group allocation (1:1) was conducted. The 10-day study period was comprised of a screening visit, a 3-day lead-in diet, a pre-diet/baseline visit including two SFA-rich high fat meals, a 7-dayPUFA-rich or a 7-day control diet, and a post-diet/final visit identical to the pre-diet/baseline visit. The experimental protocol was reviewed and approved by the Institutional Review Board (IRB). Each participant signed an informed written consent document before the start of the study.

### Subjects

Thirty-two (*n* = 16 males and n = 16 females) apparently healthy, normal weight (as determined by a body mass index of 18–24.9 kg/m^2^), and sedentary (performed < 3 h/wk. of structured exercise) men and women between the ages of 18 and 35 years were recruited and randomized to either the PUFA diet group (n = 16) or the control diet group (n = 16). Individuals with a history of chronic disease (diabetes, hyperthyroidism, hypothyroidism, cancer, or cardiovascular disease), dyslipidemia (depending on fasting blood sample collected at the screening visit), medication or supplement use other than a daily multivitamin, history of surgery or medications that would affect swallowing, digestion, absorption, or metabolism of nutrients, tobacco use, excessive weight loss (classified as > 5% of body weight in the past 3 months), anyone who had plans of becoming pregnant before or were pregnant or lactating during the study, or anyone who had plans to alter their current physical activity level or dietary habits/patterns were excluded from the study. Six participants either dropped out or were not included in the final analysis due to poor diet compliance (all in the control group). Thus, twenty-six (*n* = 13 men and n = 13 women) adults completed all study visits. All testing procedures were completed at the Human Nutrition Lab (HNL) after an 8–12 h overnight fast and an abstinence from exercise for at least 12 h.

### Screening visit

To be included in the study, participants had to have normal blood lipid profiles (fasting total cholesterol< 200 g/dL, HDL cholesterol> 40 mg/dL, LDL cholesterol< 100 mg/dL, and/or triglycerides< 150 mg/dL). The purpose of the screening visit was to exclude potential participants who were hyperlipidemic by collecting a 5 ml fasting blood sample. Further, to ensure participants were normal weight based on BMI, height and weight were measured. A stadiometer and a clinical scale were used for height and body weight measurements, respectively. Also, a 30-min resting metabolic rate (RMR) measurement was completed to determine participants’ daily energy expenditure. The blood samples were collected in vacutainers and centrifuged at 3000 rpm for 15 min at 4 °C immediately after collection. RMR (kcal/d) was measured utilizing a metabolic cart (TrueOne 2400, Parvo Medics, Sandy, UT, USA). Of the entire 30 min of RMR measurement, only the final 20 min were used to calculate RMR by the full Weir equation [30]. Estimated total daily energy needs were calculated as participant’s RMR*1.65 (based on an average United States physical activity factor) [31]. These calculations were used to estimate total daily energy needs for the 3d lead in diet, both 7d diets, and the HF meals rich in SFA. The diets were designed to maintain participants’ energy balance throughout the study. Once determined to be eligible, participants were randomly assigned to one of the two treatment conditions: PUFA diet or control diet group. Participants were blinded as to which diet they were receiving.

### Lead-in diet

For three days prior to the pre-diet visit, participants were provided with a lead-in diet that is representative of the standard American diet (Table 1). Average total daily energy needs for participants has been reported previously [29]. In the lead-in diet, approximately 29, 31, and 40% of energy was provided at breakfast, lunch, dinner + snacks, respectively. However, except for breakfast which was provided and consumed in HNL each morning, participants could choose in what order they wish to eat meals and snacks on condition that they ate all the food that was given to them each day. Participants were not permitted to consume any additional foods or caloric beverages. They also were instructed to keep a log of their food and physical activity to enhance compliance.

**Table 1:** Nutrient breakdown for each diet

Composition	Lead-in Diet	PUFA-rich Diet	Control Diet
Percentage of total energy from			
Protein	15.0	15.0	15.0
Carbohydrate	50.0	50.0	50.0
Fat	35.0	35.0	35.0
Percentage of energy from fatty acid of interest			
PUFA	7.0	21.0	7.0
n6 PUFA	7.0	12.5	7.0
n3 PUFA	0.0	8.5	0.0
SFA	13.0	5.0	13.0
MUFA	15.0	9.0	15.0

*HF* high-fat, *MUFA* monounsaturated fatty acid, *PUFA* polyunsaturated fatty acid, *SFA* saturated fatty acid, *n6 PUFA* omega 6 polyunsaturated fatty acid, *n3 PUFA* omega 3 polyunsaturated fatty acid

### Pre- and post-diet visits

At the conclusion of the 3-day lead-in diet, subjects completed a pre-diet visit where they consumed two SFA-rich, HF liquid meals: one at breakfast (0800) and one at lunch (1200). For women, the follicular phase of their menstrual cycle was appointed to complete the pre-diet visit (days 3–9 of their cycle). The participants were given 5 min to consume each liquid meal in its entirety, and the liquid meals were designed to provide 35% of the participants estimated daily energy needs (Table 2). Blood samples were taken every 30 min for 4 h post-breakfast and 4 h post-lunch. However, for this present study, the every-2-h blood samples were used for the purpose of plasma TG and ANGPTL measurements. Additionally, height and body weight were measured as previously described in the screening visit. Hip and waist circumferences were measured in triplicate using a measuring tape. The average of the three measurements was calculated and recorded as hip and waist circumferences. Body composition was measured using air displacement plethysmography (BodPod, Cosmed USA, Inc. Concord, CA, USA). After the 7-day diet, participants reported to the HNL for the post-diet visit where they consumed the same two SFA-rich, HF liquid meals and repeated the same procedures and measurements from the pre-diet visit.

**Table 2:** Nutrient breakdown for each high saturated fat test meal

Composition	Each SFA-rich HF Meal
Percentage of total daily energy	35.0
Percentage of total energy from	
Protein	5.0
Carbohydrate	25.0
Fat	70.3
Percentage of energy from fatty acids	
SFA	46.9
MUFA	15.7
PUFA	6.9

*HF* high fat, *MUFA* monounsaturated fatty acid, *PUFA* polyunsaturated fatty acid, *SFA* saturated fatty acid

### 7-day diets

At the conclusion of the pre-diet visit, participants were put on the 7-day diet (either the PUFA-rich diet or the control diet). Participants came to the HNL to consume breakfast and received the remainder of their food and beverages for the rest of the day. The percentage of energy coming from each macronutrient was identical between both the PUFA-rich diet and the control diet, providing 50% of calories from carbohydrates, 35% of calories from fat and 15% of calories from protein (Table 1). Walnuts provided a significant portion of the n3 PUFAs for the PUFA-rich diet along with salmon, tuna, flaxseed oil, canola oil, and fish oil. This amount of PUFAs consisted of ~ 3 g/day of combined EPA (2157 mg/d) and DHA (843 mg/d) (GNC Ultra Triple Strength Omega 1000 EPA & DHA, Pittsburgh, Pennsylvania, USA).

### Biochemical assays

The Wako Diagnostics L-Type Triglyceride M Assay kit (Wako Chemicals USA, Inc., Richmond, VA) was used to quantify plasma TG concentrations. Plasma glucose concentrations were measured using the glucose oxidase method. Plasma FFA concentrations were measured using the Wako Diagnostics HR Series NEFA-HR (2) assay kit (Wako Chemicals USA, Inc., Richmond, VA). Plasma ANGPTL3 and ANGPTL4 levels were measured using human ANGPTL3 and ANGPTL4 DuoSet ELISA kits (R&D Systems, Inc., Minneapolis, MN). ANGPTL8 plasma levels were measured using Betatrophin (139–198) (Human) EIA kit (Phoenix Pharmaceuticals, Inc., Burlingame, CA).

### Statistical analyses

Statistical analyses were performed using the JMP Pro 13 statistical software package. Differences in anthropometrics and fasting lipid profiles were tested within each treatment group with a paired t-test. A full factorial repeated measures ANOVA was used to test for differences in TG and ANGPTL 3, 4, and 8 concentrations for the two treatment conditions (PUFA vs. control diet) based on time, visit and sex. Additionally, mean area under the curve (AUC) difference was calculated for TG, all 3 ANGPTLs, glucose, FFA, insulin and fat oxidation for both diet groups (PUFA vs. control diet) and compared using a one-way ANOVA. Post hoc analyses were performed using a Tukey test where applicable. Statistical significance was set at *p* < 0.05, and data are presented as mean ± SD, unless otherwise specified.

## Results

Twenty-six sedentary normal weight men and women completed all study visits and were included in the final study analysis (PUFA-diet: *n* = 8 women and n = 8 men; control diet: *n* = 5 women and n = 5 men) (Table 3**,** Additional file 1 CONSORT flow diagram in supplementary material). In the PUFA-rich diet group, there were no changes in anthropometric and blood pressure measurements between pre- and post-diet visits in either the male or female participants. A significant decrease in weight and BMI was seen for both males and females in the control diet group from pre- to post-diet. There was also a decrease in hip circumference in control females between study visits. As reported previously, there were significant decreases in fasting total cholesterol, TG, non-HDL, LDL cholesterol, VLDL cholesterol, and cholesterol/HDL ratio from pre- to post- diet visits in the PUFA-rich diet group, while only the TG and LDL cholesterol levels decreased in the control group from pre- to post-diet visits [29]. In the present study, these changes were in both the male (*p* < 0.05) and female (*p* < 0.01) participants in the PUFA-rich diet group, but not in the control group.

**Table 3:** Participant Characteristics, Fasting Lipids and ANGPTL Levels (prior to SFA-rich meal consumption)

	PUFA-rich diet (*n* = 16)				Control diet (*n* = 10)			
	Males (n = 8)		Females (n = 8)		Males (n = 5)		Females (n = 5)	
	Pre-diet	Post-diet	Pre-diet	Post-diet	Pre-diet	Post-diet	Pre-diet	Post-diet
Age (year)	23.3 ± 5.3	–	23.4 ± 3.9	–	20.2 ± 1.3	–	22.8 ± 6.4	–
Height (cm)	177.2 ± 9.7	–	166.3 ± 10.7	–	182.6 ± 7.0	–	165.8 ± 4.0	–
Weight (kg)	72.9 ± 7.2	72.5 ± 7.4	59.9 ± 10.9	60.3 ± 10.5	73.2 ± 9.8	72.8 ± 9.9*	60.1 ± 7.5	59.5 ± 7.7*
BMI (kg/m^2^)	23.3 ± 2.2	23.1 ± 2.2	21.6 ± 2.7	21.7 ± 2.7	21.9 ± 2.2	21.8 ± 2.2*	21.8 ± 2.3	21.6 ± 2.3*
Body Fat %	17.5 ± 7.0	17.0 ± 7.4	25.7 ± 5.9	26.6 ± 6.5	21.9 ± 6.1	21.3 ± 7.2	30.8 ± 5.5	30.1 ± 6.4
WC (cm)	81.8 ± 5.4	81.4 ± 6.2	71.1 ± 5.1	70.5 ± 5.5	82.7 ± 6.3	82.1 ± 6.9	73.6 ± 3.6	72.6 ± 4.4
HC (cm)	96.5 ± 4.0	96.1 ± 3.8	96.5 ± 5.7	96.9 ± 5.4	100.0 ± 4.2	98.6 ± 7.1	100.1 ± 7.7	99.1 ± 8.3*
WHR	0.8 ± 0.04	0.8 ± 0.04	0.7 ± 0.06	0.7 ± 0.03	0.8 ± 0.05	0.8 ± 0.04	0.7 ± 0.03	0.7 ± 0.02
SBP (mmHg)	116.7 ± 12.9	119.6 ± 12.5	108.6 ± 9.5	102.7 ± 7.0	112.1 ± 9.0	113.6 ± 12.4	107.9 ± 8.8	104.9 ± 8.8
DBP (mmHg)	69.1 ± 8.7	71.5 ± 8.3	68.9 ± 4.1	64.2 ± 5.7	70.9 ± 7.5	72.3 ± 4.7	70.1 ± 3.8	71.9 ± 8.7
Cholesterol (mg/dL)	138.9 ± 23.5	112.3 ± 16.5*	158.1 ± 22.6	124.4 ± 16.4*	149.8 ± 18.4	138.8 ± 15.2*	146 ± 29.1	146.8 ± 27.7
HDL-cholesterol (mg/dL)	43.4 ± 8.4	44 ± 8.3	52.4 ± 14.1	51.9 ± 10.3	40.4 ± 3.9	41.8 ± 9.3	55.6 ± 14.1	55.4 ± 13.9
Non-HDL (mg/dL)	95.5 ± 22.7	68.3 ± 18.6*	105.8 ± 16.7	71.3 ± 14.7*	109.4 ± 18.2	97 ± 17.5*	90.4 ± 19.6	91.4 ± 16.8
LDL cholesterol (mg/dL)	79.8 ± 23.8	52.4 ± 21.2*	91.8 ± 17.9	57.9 ± 13.6*	98.2 ± 20.0	85.2 ± 17.4*	78.8 ± 19.5	76.4 ± 17.7
VLDL cholesterol (mg/dL)	13.4 ± 5.0	9.6 ± 5.7*	14.8 ± 2.1	9.7 ± 1.9*	14.8 ± 4.5	12.2 ± 1.6	12.8 ± 3.1	11.4 ± 2.8
Chol/HDL Ratio	3.3 ± 0.81	2.6 ± 0.7*	3.1 ± 0.6	2.5 ± 0.4*	3.7 ± 0.6	3.4 ± 0.8	2.7 ± 0.3	2.7 ± 0.4
Triglycerides (mg/dL)	66.4 ± 24.9	48.3 ± 28.4*	74.1 ± 10.5	48.8 ± 10.0*	74.4 ± 21.5	60.8 ± 9.8	64 ± 14.5	58.2 ± 13.5
ANGPTL3 (pg/mL)	240.6 ± 16.2	203.5 ± 20.7	236.6 ± 20.5	194.6 ± 17.0*	243.7 ± 46.1	248.5 ± 32.0	250.2 ± 33.3	238.8 ± 17.6
ANGPTL8 (ng/mL)	1.20 ± 0.21	1.15 ± 0.25	0.99 ± 0.12	0.59 ± 0.07*	1.7 ± 0.7	2.3 ± 1.3	1.5 ± 0.5	2.1 ± 0.9
ANGPTL4 (ng/mL)	22.9 ± 2.4	32.2 ± 4.1	28.0 ± 9.8	33.4 ± 7.6	36.5 ± 15.2	36.6 ± 8.2	21.4 ± 5.8	24.8 ± 6.8

Values are presented as Mean ± SD

*BMI* body mass index, *WC* waist circumference, *HC* hip circumference, *WHR* waist-to-hip ratio, *SBP* systolic blood pressure, *DBP* diastolic blood pressure, *HDL* high-density lipoprotein, *LDL* low-density lipoprotein, *VLDL* very low-density lipoprotein, *ANGPTL* Angiopoietin-Like Protein

*Indicates significant difference from pre- to post- diet, *p* < 0.05

### Triglyceride responses

**Fig. 1:**
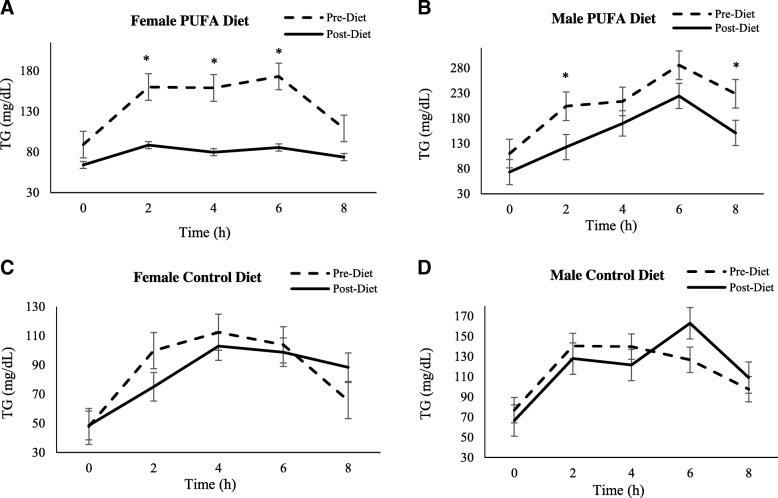
Plasma TG response to SFA-rich meals before and after the PUFA diet. Changes in plasma TG are presented in PUFA-diet female (**a**) (*n* = 8) and male (**b**) (*n* = 8) subjects, and control diet female (**c**) (*n* = 5) and male (D) (*n* = 5) subjects before (dashed line) and after (solid line) the diets. In PUFA-diet females only, plasma TG concentrations were significantly lower at 2, 4, and 6 h after the 7-day diet. These differences were not significant in PUFA males or either sexes in the control group; * indicates a significant decrease compared to pre-diet levels in females (*p* < 0.05)

**Fig. 2:**
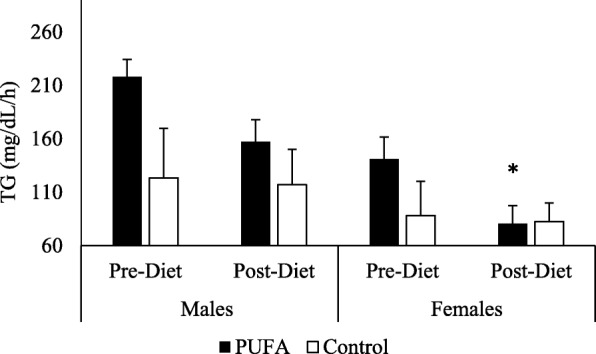
Plasma TG Area Under the Curve (AUC). Changes from fasting through 8 h post meals were assessed using AUC in females and males on PUFA-rich and control diets. The 8-h AUC indicated that the female PUFA-diet group reduced total TG response whereas the other 3 groups did not; * indicates a significant decrease compared to pre-diet levels in females (*p* < 0.05)

Fasting TG concentrations (prior to high-SFA meal consumption) were significantly lower at the post-diet vs. the pre-diet visit for all subjects combined and when analyzed by sex for the PUFA-rich diet (p < 0.05). No changes were observed in the control group (Table 3). Aside from fasting TG concentrations, there was an effect of sex on the SFA-rich meal responses (after consumption of SFA-rich meals) (p < 0.05). Therefore, data were presented and analyzed by sex. For the SFA-rich meal responses in females, there was a significant main effect for visit (*p* < 0.001) and time (p < 0.001), a trend for a treatment effect (*p* = 0.08) and a significant visit by time interaction (*p* = 0.01) (Fig. 1a). Post-hoc analyses revealed that TG concentrations were significantly lower in the PUFA group at 2, 4, and 6 h postprandial time-points after the 7-day diet. AUC values were 141.2 mg/dL/h (SD 18.7) vs. 80.7 mg/dL/h (SD 6.5), p = 0.01, for pre- vs. post-diet, respectively). There were no significant differences from pre- to post-diet for females in the control diet group (88.2 mg/dL/h (SD 10.6) vs. 82.5 mg/dl/8 h (SD 6.3), ns, for pre- vs. post-diet, respectively) (Fig. 2).

For male participants, there was a significant main effect of time (*p* < 0.001) and visit (*p* = 0.02) but no treatment effects or interactions for the SFA-rich meal challenge. The visit effect was for lower TG following the 7-day diets for the combined diets (Fig. 1b). For the PUFA diet alone, there was a trend for lower plasma TG concentrations (218.2 mg/dL/h (SD 38.1) vs. 157.3 mg/dL/h (SD 23.7), *p* = 0.09, for pre- vs. post-diet, respectively) after the diet but no change in the control group (123.5 mg/dL/h (SD 13.4) vs. 117.0 mg/dL/h (SD 10.9), ns, for pre- vs. post-diet, respectively) (Fig. 2).

### ANGPTL results

**Fig. 3:**
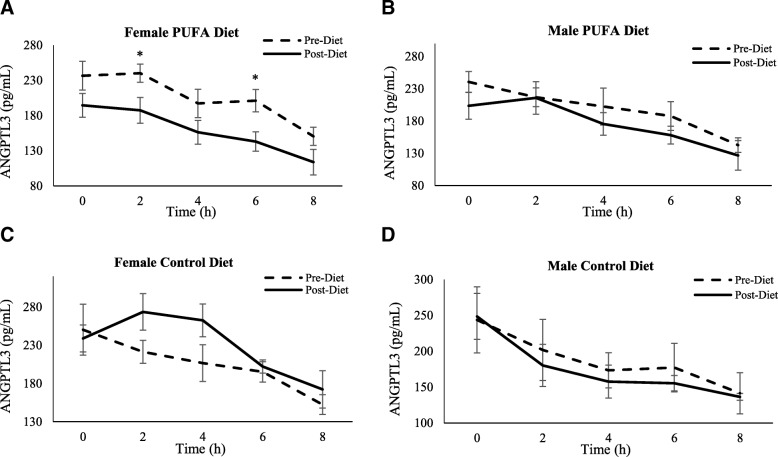
Plasma ANGPTL3 response to SFA-rich meals before and after the PUFA diet. Changes in plasma ANGPTL3 are presented in PUFA-diet female (**a**) (*n* = 8) and male (**b**) (*n* = 8) subjects, and control diet female (**c**) (*n* = 5) and male (**d**) (*n* = 5) subjects before (dashed line) and after (solid line) the diets. In PUFA-diet females only, fasting ANGPTL3 values decreased from pre- to post-diet visit (*p* < 0.01), but not in PUFA males. There was no significant change seen from pre- to post-diet visit in either sexes in the control group; * indicates a significant decrease compared to pre-diet levels in females (*p* < 0.05)

*ANGPTL3 -* The repeated measures ANOVA revealed a significant effect of treatment (*p* < 0.001), visit (*p* = 0.01), time (*p* = 0.03) and sex (*p* = 0.02), as well as a treatment by visit interaction (*p* = 0.01) on ANGPTL3 concentrations (Fig. 3a-b). Since there was an effect of sex on the SFA-rich meal responses, both fasting and postprandial ANGPTL data were presented and analyzed by sex. In females in the PUFA group, we found that fasting ANGPTL3 values decreased from pre- to post-diet visit (236.6 pg/mL (SD 20.5) vs. 194.6 pg/mL (SD 17.0), *p* < 0.01, for pre- and post-diet visits, respectively), but not in PUFA males (240.6 pg/mL (SD 16.2) vs. 203.5 pg/mL (SD 20.7), ns; for pre- and post-diet, respectively). There was no significant change seen from pre- to post-diet visit in either sexes in the control group (Table 3).

**Fig. 4:**
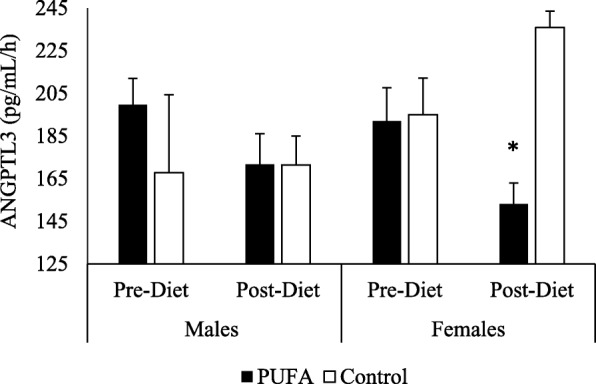
Postprandial ANGPTL3 Area Under the Curve (AUC). Changes from fasting through 8 h post meals were assessed using AUC in females and males on PUFA-rich and control diets. The 8-h AUC indicated that the female PUFA-diet group reduced total ANGPTL3 response whereas the other 3 groups did not; * indicates a significant decrease compared to pre-diet levels in females (*p* < 0.05)

In response to the SFA-rich meal, the PUFA diet group showed a significant reduction in post-meal ANGPTL3 levels in females (AUC: 192.0 pg/mL/h (SD 14.4) vs. 153.2 pg/mL/h (SD 9.8), *p* = 0.02; for pre- vs. post-diet, respectively), but not in males (AUC: 199.6 pg/mL/h (SD 12.4) vs. 171.6 pg/mL/h (SD 15.5), ns; for pre- vs. post-diet, respectively). In the control group, there were no significant changes from pre- to post-diet visit in either sex (Figs. 3c, d and fig. 4).

**Fig. 5:**
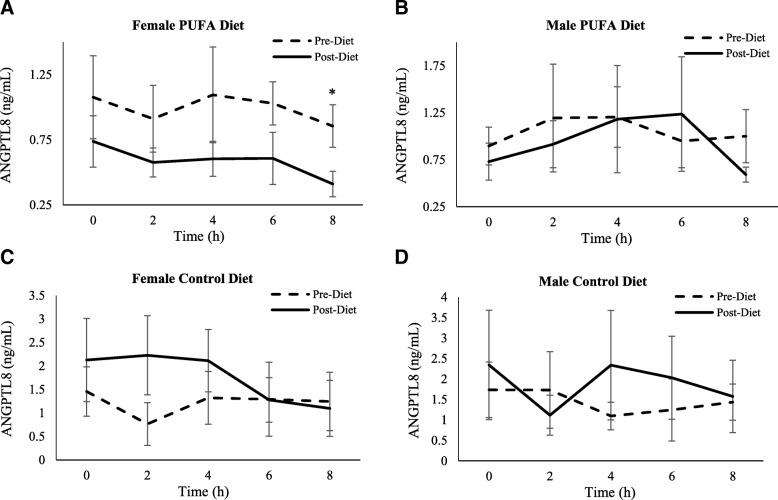
Plasma ANGPTL8 response to SFA-rich meals before and after the PUFA diet. Changes in plasma ANGPTL8 are presented in PUFA-diet female (**a**) (*n* = 8) and male (**b**) (*n* = 8) subjects, and control diet female (**c**) (*n* = 5) and male (**d**) (*n* = 5) subjects before (dashed line) and after (solid line) the diets. In PUFA-diet females only, fasting ANGPTL8 values decreased from pre- to post-diet visit (*p* < 0.05), but not in PUFA males. There was no significant change seen from pre- to post-diet visit in either sexes in the control group; * indicates a significant decrease compared to pre-diet levels in females (*p* < 0.05)

*ANGPTL8 –* Similar to the ANGPTL3 results, the ANOVA revealed a significant effect of treatment (*p* = 0.01), time (*p* = 0.04) and sex (*p* = 0.02), as well as an interaction effect of visit by treatment (*p* = 0.03) (Fig. 5a-b). When analyzed by sex, fasting ANGPTL8 values decreased from pre- to post-diet visit for PUFA females (0.99 ng/mL (SD 0.12) vs. 0.59 ng/mL (SD 0.07), p = 0.04, for pre- and post-diet visits, respectively), but not in PUFA males (1.20 ng/mL (SD 0.21) vs. 1.15 ng/mL (SD 0.25), ns; for pre- and post-diet, respectively). There is no change from pre- to post-diet visit in either sex in the control group (Table 3).

**Fig. 6:**
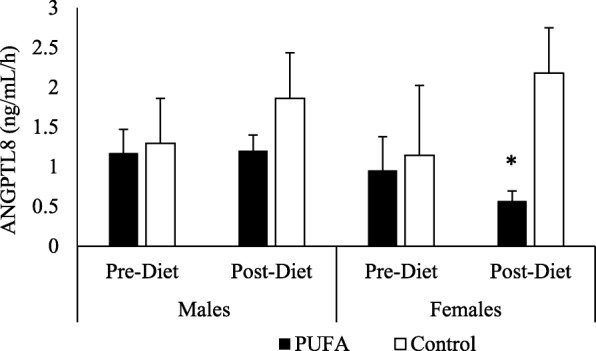
Postprandial ANGPTL8 Area Under the Curve (AUC). Changes from fasting through 8 h post meals were assessed using AUC in females and males on PUFA-rich and control diets. The 8-h AUC indicated that the female PUFA-diet group reduced total ANGPTL8 response whereas the other 3 groups did not; * indicates a significant decrease compared to pre-diet levels in females (*p* < 0.05)

In response to the SFA-rich meal, the PUFA group showed significantly lower post-meal ANGPTL8 levels in females (AUC: 0.95 ng/mL/h (SD 0.20) vs. 0.57 ng/mL/h (SD 0.12), *p* = 0.04; for pre- vs. post-diet, respectively) but not in males (AUC: 1.17 ng/mL/h (SD 0.30) vs. 1.20 ng/mL/h (SD 0.42), ns; for pre- vs. post-diet, respectively). There is no such change from pre- to post-diet visit in either sex in the control group (Figs. 5c, d and 6).

**Fig. 7:**
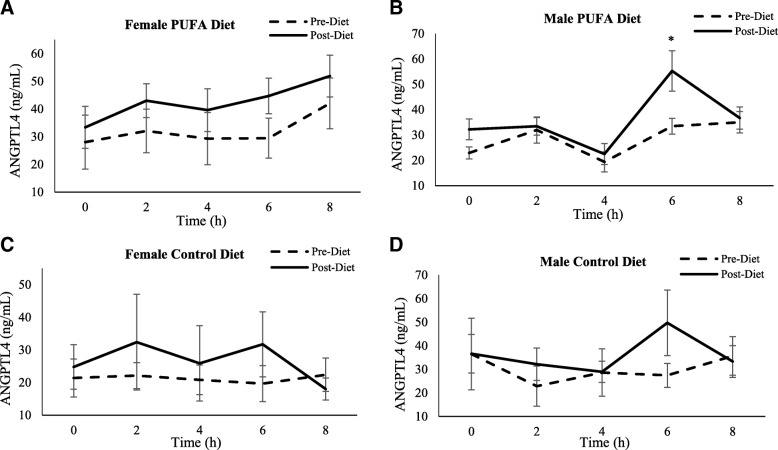
Plasma ANGPTL4 response to SFA-rich meals before and after the PUFA diet. Changes in plasma ANGPTL4 are presented in PUFA-diet female (**a**) (*n* = 8) and male (**b**) (*n* = 8) subjects, and control diet female (**c**) (*n* = 5) and male (**d**) (*n* = 5) subjects before (dashed line) and after (solid line) the diets. There were no significant changes in ANGPTL4 from pre- to post-diet visit in either sexes or in either diet group

**Fig. 8:**
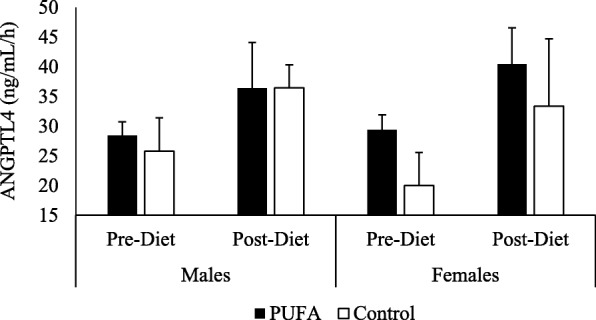
Postprandial ANGPTL4 Area Under the Curve (AUC). Changes from fasting through 8 h post meals were assessed using AUC in females and males on PUFA-rich and control diets. There were no differences in 8-h AUC between diets among any of the four groups

*ANGPTL4* **–** Unlike ANGPTL-3 and -8, there were no differences for ANGPTL4 fasting concentrations between the study groups or the two sex at either visit (Table 3). For the SFA meal response, there was a significant visit effect (*p* = 0.01) on ANGPTL4 concentrations (Fig. 7a-d). The diets showed a significant increase in post-meal ANGPTL4 levels from pre- to post-diet (AUC: 28.9 ng/mL/h (SD 3.9) vs. 38.4 ng/mL/h (SD 3.3), p = 0.04; for pre- vs. post-PUFA diet, respectively; and 22.9 ng/mL/h (SD 3.4) vs. 34.9 ng/mL/h (SD 6.0) for pre- and post-control diet, respectively). However, there were no differences between diet groups at either pre- or post-diet visits. Also, males and females did not respond differently to SFA-rich meals (Fig. 8).

## Discussion

The aim of this study was to determine whether a high-PUFA diet could mitigate the effects of a high-fatSFA-rich meal by reducing postprandial TG levels. Research suggests that both the amount and type of dietary fat influences postprandial lipemia, showing that test meals containing up to 50 g of fat can substantially increase TG concentrations [32]. Furthermore, previous studies have reported reduced postprandial TG concentrations in response to n-3 PUFA consumption, while SFA consumption leads to increased postprandial TG concentrations [33, 34]. However, to our knowledge no study has utilized a whole-food based, longer-term PUFA diet to measure postprandial TG response to high fat meals rich in SFA. Therefore, for the first time, we are showing that a PUFA-rich diet reduces postprandial TG concentrations in females, following a SFA-rich meal. While this study design is unique, our results are consistent with previous literature showing that postprandial TG concentrations are lower after consuming either a PUFA or SFA meal while following a PUFA background diet compared to a SFA background diet [35]. These findings suggest that a diet rich in PUFAs may provide metabolic protection from high-fat SFA-rich meals. Also, since the pre and post-diet high-SFA meals were of identical composition, the differences observed from pre to post-diet intervention are due to the chronic effect of the 7 days of high PUFA consumption.

Furthermore, the current study demonstrates that ANGPTL3 and − 8 are both decreased in females following a high PUFA diet. ANGPTL8 has been reported to downregulate adipose triglyceride lipase (ATGL) that catalyzes the initial step in hydrolyzing triglyceride in adipocytes, hence plays an important role in degrading lipid droplet/adipose in mammalian cells [36–39]. Several studies have shown postprandial effects on all three ANGPTLs, but thus far, no studies have demonstrated a diet-induced effect on ANGPTL3, − 4, or − 8. One previous study compared tissue-specific uptake of dietary fat in male versus female wild-type and Angptl4−/− mice and reported no sex differences [40], which is in agreement with our human data in the baseline measures. However, our study looked at the impact of a high PUFA diet in males and females independently and found that only females displayed a diet-induced change in ANGPTL3, and − 8. From these data, it is worth assessing whether the presence of estrogen or the absence of androgens may influence ANGPTL3 or − 8 activity in females, especially pre- versus post-menopausal. While there was no observed change in ANGPTL4, it is possible that cleaved versus N-terminal forms of the protein were differentially affected by diet. Therefore, further research is needed to separately investigate different forms of the protein since the ELISA-based assay that we used to measure ANGPTL4 could not differentiate between the cleaved and the N-terminal form of this protein. Moreover, ANGPTL4 is thought to be associated with HDL in plasma [41] and our measurements did not distinguish between HDL-associated and HDL-free ANGPTL4. There is limited evidence in the literature on the complex functionality of ANGPTL4 in response to diet. Thus, it is possible that ANGPTL4 may differ by diet, form of the protein and differential HDL association, although it would require a more detailed analysis of its status in various plasma fractions to be sure.

The lack of reduction in TG concentrations from pre- to post-diet in the male PUFA diet group participants was unexpected. Although this was not a mechanistic study, and we do not know the exact mechanism by which PUFAs reduce postprandial TG concentrations differently for males and females. There are noticeable physiological differences between males and females and sex as a biological variable (SABV) should be accounted for in basic and clinical research [42]. In looking at the differences between the way males and females metabolize and store fat, previous research is consistent with our results in finding that males and females do not respond to meal ingestions in the same way [43]. It is believed that the gonadal hormones, estrogen and androgen, are responsible for the differences in energy balance and meal response between sexes [44]. Specifically, studies suggest that estradiol suppresses free fatty acid and TG synthesis and accumulation in the blood and tissues, and increases fat oxidation in females, compared to males. Some studies have also shown that females are more resistant to metabolic disturbances caused by high-fat diets compared to males due to the presence and abundance of estrogen receptor-α (ERα) and estrogen receptor-β (ERβ) [44–46]. ERα is the main receptor in hepatocytes and in conjunction with the presence of estradiol, they control genes involved in glucose, lipid, protein and cholesterol homeostasis [46].Taken together, these findings might help explain the differences seen in TG concentrations in our study between the male and female participants. A longer period study would be beneficial to highlight more prominent gender specific differences if any, in order to deduce more physiologically meaningful conclusions and comparisons. Finally, we acknowledged that the reduction in body weight occurred in the control group from pre- to post-diet visit, however it is likely to be clinically non-significant. In spite of our internal speculations about the cause for this, we cannot say with certainty how or why this happened. Future studies on longer term interventions might provide more accurate information on the effects of different dietary fatty acids on body weight changes.

## Conclusions

Altogether, the results of this study indicate that consuming a high PUFA diet is capable of reducing the detrimental effects of high SFA meals. Both males and females improved plasma TG levels, albeit to a far greater extent in females. Further studies are needed to assess whether the presence or absence of estrogen or testosterone are responsible for these effects and to what extent PUFA intake can be reduced while still providing protection. Finally, these results provide further support for the role of SABV in physiology and should be included in study design when feasible.

## Additional file

Additional file 1: CONSORT flow diagram (DOC 33 kb)

## Abbreviations

ANGPTL: Angiopoietin-like proteins
ATGL: Adipose Triglyceride Lipase
AUC: Area Under the Curve
CVD: Cardiovascular Disease
DHA: Docosahexaenoic Acid
EPA: Eicosapentaenoic Acid
ERα: Estrogen Receptor-α
ERβ: Estrogen Receptor-β
FFA: Free Fatty Acid
HDL: High Density Lipoprotein
HF: High Fat
HNL: Human Nutrition Lab
IRB: Institutional Review Board
LDL: Low Density Lipoprotein
LPL: Lipoprotein Lipase
PPAR: Peroxisome-Proliferator-Activated Receptor
PUFA: Polyunsaturated fatty acids
RMR: Resting Metabolic Rate
SABV: Sex As a Biological Variable
SFA: Saturated Fatty Acids
TG: Triglyceride
VLDL: Very Low-Density Lipoprotein
