# Supplementary material for: A 7-day high-PUFA diet reduces angiopoietin-like protein 3 and 8 responses and postprandial triglyceride levels in healthy females but not males: a randomized control trial
Source: BMC Nutr. 2019 Jan 6;5:1. doi: 10.1186/s40795-018-0262-7 (PMC7050740; doi:10.1186/s40795-018-0262-7)
Supplement: Supplementary file 1 — CONSORT flow diagram (DOC 33 kb) [file 40795_2018_262_MOESM1_ESM.doc]

**CONSORT 2010 Flow Diagram**

**Allocation**

**Analysis**

**Follow-Up**

**Enrollment**

Assessed for eligibility (n= 45)

Excluded (n= 13)

♦  Not meeting inclusion criteria (n= 9)

♦  Declined to participate (n= 4)

♦  Other reasons (n= 0)

Analysed (n= 16)
♦ Excluded from analysis (n= 0)

Lost to follow-up (n= 0)

Discontinued intervention (n= 0)

Allocated to PUDA diet group (n= 16)

♦ Received allocated intervention (n= 16)

♦ Did not receive allocated intervention (n= 0)

Lost to follow-up (n=0)

Discontinued intervention (n= 6)

♦ Drop-out (n= 4)

♦ Excluded due to poor compliance (n= 2)

Allocated to control diet group (n= 16)

♦ Received allocated intervention (n= 16)

♦ Did not receive allocated intervention (n= 0)

Analysed (n= 10)
♦ Excluded from analysis (n= 0)

Randomized (n= 32)
